# Clinicopathologic Features of Cutaneous Malignant Melanoma and Their Impact on Prognosis

**DOI:** 10.7759/cureus.10450

**Published:** 2020-09-14

**Authors:** Usman Atique, Sajid Mushtaq, Iftikhar Ali Rana, Usman Hassan

**Affiliations:** 1 Histopathology, Shaukat Khanum Memorial Cancer Hospital and Research Centre, Lahore, PAK; 2 Pathology, Shaukat Khanum Memorial Cancer Hospital and Research Centre, Lahore, PAK

**Keywords:** melanoma, prognosis, breslow, metastasis, clark

## Abstract

Introduction: Melanoma ranks 19th among malignancies overall and second among cutaneous types. The incidence worldwide has been on the rise over the last seven decades. Various prognostic factors have been assessed and found to have a profound impact on patient outcome. However, no such studies have been attempted in our population. Our study aimed to have an insight into the behavior of malignant melanoma in our population.

Materials and Methods: Cases of cutaneous malignant melanoma treated and followed up at our institute were included in this study. Cases of mucosal and choroidal melanoma were excluded. The parameters noted were age, gender, tumor thickness, Clark level, and presence of ulceration. These parameters were individually correlated with development of distant metastasis, two-year survival, survival duration, and primary tumor and lymph node stage. Appropriate statistical analyses were done.

Results: Thirty patients of cutaneous malignant melanomas were treated and followed up at our institution. There was male predilection of 1:1.5. Mean age at diagnosis was 50.1 years. Two-year survival was significantly better in females. Sun-exposed areas of the skin were most commonly involved followed by anal canal that has an unusually high incidence in our society. Majority of our cases were pT4(25) on tumor, nodal status, metastasis (TNM) staging at time of diagnosis. Increasing tumor thickness in terms of primary tumor staging was not found to have any significant impact on two-year survival, distant metastasis, lymph node stage, or survival duration. Sixty percent of cases had ulceration. There was no statistically significant effect on two-year survival (78% in ulcerated group vs 75% in nonulcerated group) and distant metastasis (61% vs 58.3%). In terms of Clark level, 20 cases were level V, seven cases were level IV, two were level III, and one was level I. There was no statistically significant difference between the Clark levels in terms of two years survival, development of distant metastasis, and lymph node stages.

Conclusion: Melanoma is an aggressive malignancy that causes high morbidity and morality. It commonly presents at an advanced stage at time of diagnosis in our population. Broader studies are required with early-stage melanomas to compare the various prognostic factors and their impact on prognosis.

## Introduction

Malignant melanoma is the 19th most common malignancy worldwide, with cutaneous malignant melanoma accounting for 90% of cases. There is considerable global variation in incidence, with the highest in Australia (37 per 100,000) and the lowest in South and Central Asia (0.2 per 100,000) [1]. This trend is attributed to racial skin phenotype as well as sun exposure, with 98.2% of affected individuals in the United States being fair-skinned. Over the past few decades, the incidence of melanoma in the United States has increased from 1 per 100,000 in 1940 to 17.74 per 100,000 in 2000. Other regions, such as Europe, Australia, and Canada, have also witnessed a marked increase in incidence [2]. The American Cancer Society estimates that approximately 6,850 deaths from malignant melanoma will occur in the United States in 2020 [3].

Various prognostic factors have been studied and are included in the routine reporting of malignant melanoma. The College of American Pathologists (CAP) includes procedure, anatomical site, melanoma subtype, tumor thickness (Breslow thickness), Clarks level, tumor infiltrating lymphocytes, tumor regression, neurotropism, lymphovascular invasion, microsatellites, and presence of nontraumatic ulceration as the required reporting parameters of prognosis in the report of an excision specimen [4]. 

In a study involving 17,600 cases of malignant melanoma, Balch et al. found that tumor thickness and presence of ulceration were the most significant independent predictors of survival in localized disease [5]. They found that among thin melanomas, the 10-year survival rate was significantly lower in ulcerated lesions (76% versus 86%). The incidence of ulceration increased with tumor thickness. Among patients with nodal metastasis, the number of metastatic lymph nodes and the presence of ulceration were the most important factors. In cases involving metastasis, the presence of non-visceral metastasis had better prognosis than visceral metastasis, with a statistically significant difference in one-year survival but no significant difference in two-year survival.

The anatomical Clark level remains an independent predictor of prognosis; however, due to its poor reproducibility, it is included as an optional data item in the CAP reporting guidelines [4]. The impact of tumor regression has been debated over time, with numerous studies reporting variable results. Zugne et al. found improved survival in patients exhibiting tumor regression [6]. To our knowledge, however, no studies have investigated the prognosis of malignant melanoma in our population. Accordingly, our aim was to assess the major clinicopathological factors of malignant melanoma and their effect on prognosis and disease outcome.

## Materials and methods

This was a retrospective study. The study sample included all patients diagnosed with malignant melanoma and treated at Shaukat Khanum Memorial Cancer Hospital with follow-up records of at least two years. Patients with choroidal melanomas and those who were lost to follow-up were excluded from this study. The cases were retrieved from the hospital information system along with the reports. Histopathology slides were reviewed by a consultant histopathologist and a resident histopathologist. The following parameters were noted: age, sex, tumor size, site, presence of ulceration, maximum tumor thickness (Breslow thickness), mitotic rate, Clark level, lymphovascular invasion, and primary tumor and lymph node staging using tumor, nodal status, metastasis (TNM) eighth edition. Patient follow-up notes were examined for development of distant metastasis and overall survival duration. The information was noted on a research proforma.

Data analysis was performed using SPSS version 20 (IBM Corporation, Armonk, NY). Qualitative variables, including the presence of metastasis, tumor site, Clark level, presence of ulceration, lymphovascular invasion, and primary tumor and lymph node staging (using using eighth edition TNM system), are expressed as frequencies and percentages. Quantitative variables, including tumor thickness, mitotic rate, and age, are expressed as range and mean ± two standard deviations (SD).

The chi-squared test was used to determine possible associations between qualitative (ulceration, lymphovascular ulceration, lymphovascular invasion, Clark levels) variables with prognostic parameters (two-year survival, presence of distant metastasis, primary tumor, and lymph node staging). The independent samples t-test was used to determine the effect of quantitative variables, including tumor thickness and number of mitoses on survival duration, two-year survival, and the development of distant metastasis. P value of ≤0.05 was taken as significant.

## Results

The present study included 30 (18 male, 12 female) patients who were treated and followed up at the Shaukat Khanum Memorial Cancer Hospital and Research Centre. The age of the subjects ranged from 23 to 71 years (23 to 71 years in males, 30 to 58 years in females). Mean (± SD) age at diagnosis was 50.1 years (47.8 ± 8.1 years in males, 51.3 ± 13.7 years in females). Among males, tumor size ranged from 0.9 to 10.0 cm, with a mean of 3.5 ± 2.3 cm. Tumor size in females ranged from 0.9 to 14.0 cm, with a mean of 4.4 ± 3.78 cm. The depth of invasion was 0 to 4.5 cm in males and 0.4 to 12 cm in females (mean depth 1.8 ± 1.32 cm in males, 1.6 ± 3.24 cm in females). The frequency of distant metastasis was 58.3% (7/12) in females and 61% in males (11/18). This difference was not statistically significant according to chi-squared test results (p > 0.05). The two-year survival was 83% (10/12) and 72% (13/18) in males and females, respectively. The chi-squared test revealed that the difference in two-year survival was statistically significant. The overall survival duration was 9 to 74 months in males (mean: 42.3 ± 18.4 months) and 31 to 87 months in females (mean, 48 ± 16.2 months). The independent samples t-test revealed no statistically significant overall survival difference in both sexes. On pT staging, 9/12 (75%) females and 16/18 (89%) males had pT4 disease at time of diagnosis. Among them, six males and six females exhibited no lymph node metastasis. Six males and two females were N1. Three males and three females had N2 disease, and three males and one female exhibited N3. The chi-squared test did not reveal any statistically significant difference in primary tumor and lymph node staging in both sexes.

The most commonly involved site was the skin of the limbs (n = 13) followed by the anal canal (n = 11); six cases were from chest wall and head and neck region. 

 Based on tumor thickness, four groups were formed using the TNM staging system (eighth edition). Twenty-five cases (83.3%) had T4 disease (>4 mm), four were pT3 (2-4 mm), and one case was T1 (<1 mm). The effects of tumor thickness on two-year survival, lymph node staging, and development of distant metastasis are summarized in Table 1. There was no statistically significant effect of tumor thickness level on two-year survival, lymph node stage, and distant metastasis. Overall survival was 9 to 74 months in T4 disease (mean, 45 months) and 34 to 87 months in T3 (mean, 47 months) (Table 1).

**Table 1:** Effect of tumor stage on prognostic parameters

Primary tumor stage (n = 30)	Lymph node stage					Two-year survival			Distant metastasis		
	N0	N1	N2	N3	p value	Alive	Dead	p value	Present	Absent	p value
pT1	0	1	0	0	0.519	1	0	0.217	0	1	0.567
pT3	2	2	0	0		4	0		2	2	
pT4	10	5	6	4		19	6		16	9	

**Figure 1:**
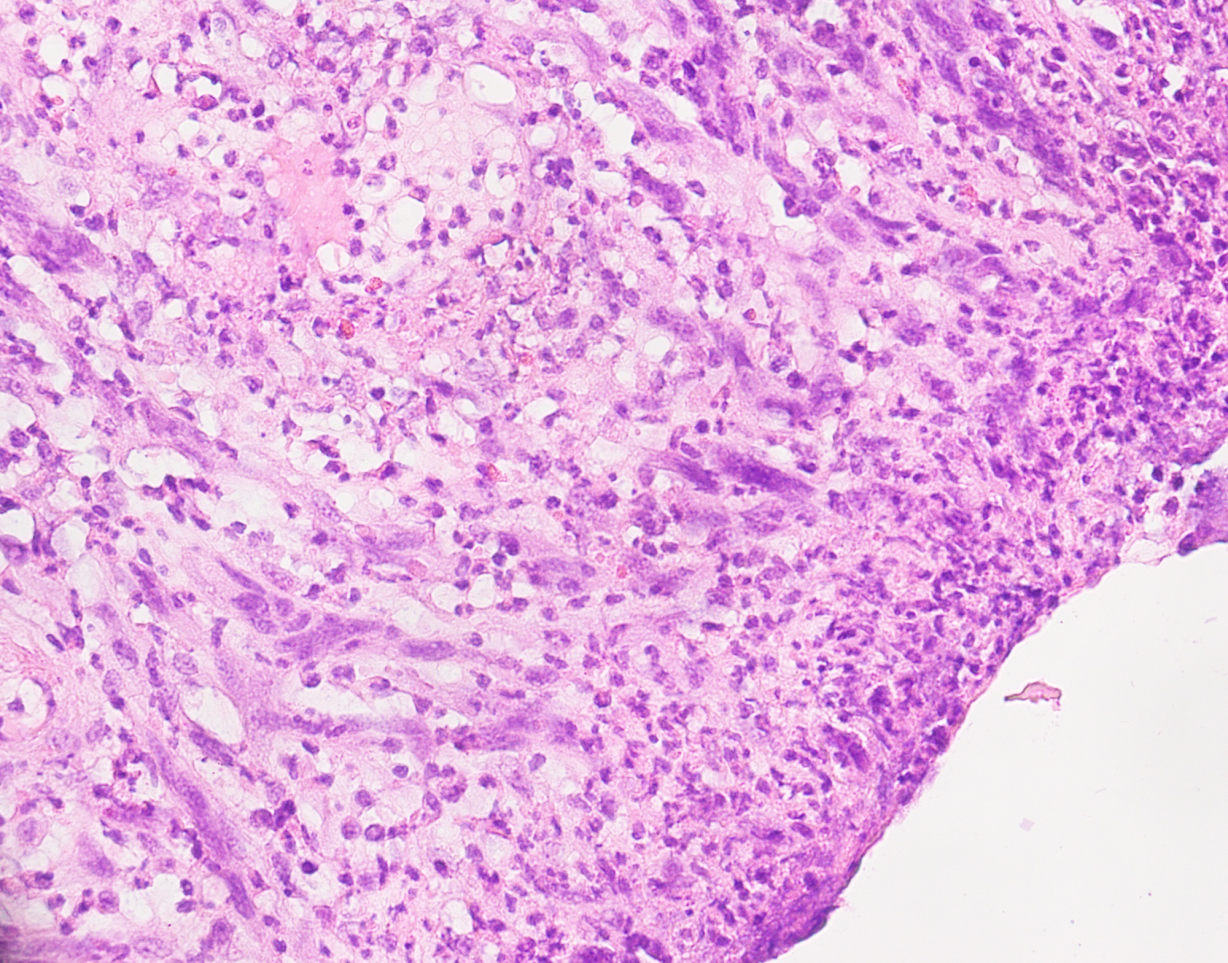
Surface ulceration in malignant melanoma; an important prognostic feature.

Eighteen patients exhibited nontraumatic ulceration (60%) (Figure 1), 17 of whom were found to have pT4 disease (eight in the nonulcerated group). One case had pT3 disease (three in the nonulcerated group), and no ulcerated case had pT1 disease. The effects of ulceration on two-year survival, lymph node stage, and distant metastasis are summarized in Table 2. Survival ranged from 9 to 60 months in cases with ulceration (mean, 44 months) and 25 to 87 months in nonulcerated cases (mean, 48 months). There was no statistically significant difference between ulcerated and nonulcerated cases in terms of distant metastasis, two-year survival, lymph node and tumor stage, and survival duration (Table 2) (Figure 1).

**Table 2:** Effect of ulceration on outcome in malignant melanoma

Presence of ulceration (n = 30)	Lymph node stage					Two-year survival			Distant metastasis		
	N0	N1	N2	N3	p value	Alive	Dead	p value	Present	Absent	p value
Present	8	3	4	3	0.584	14	4	0.956	11	7	0.588
Absent	4	5	2	1		9	3		7	5	

**Figure 2:**
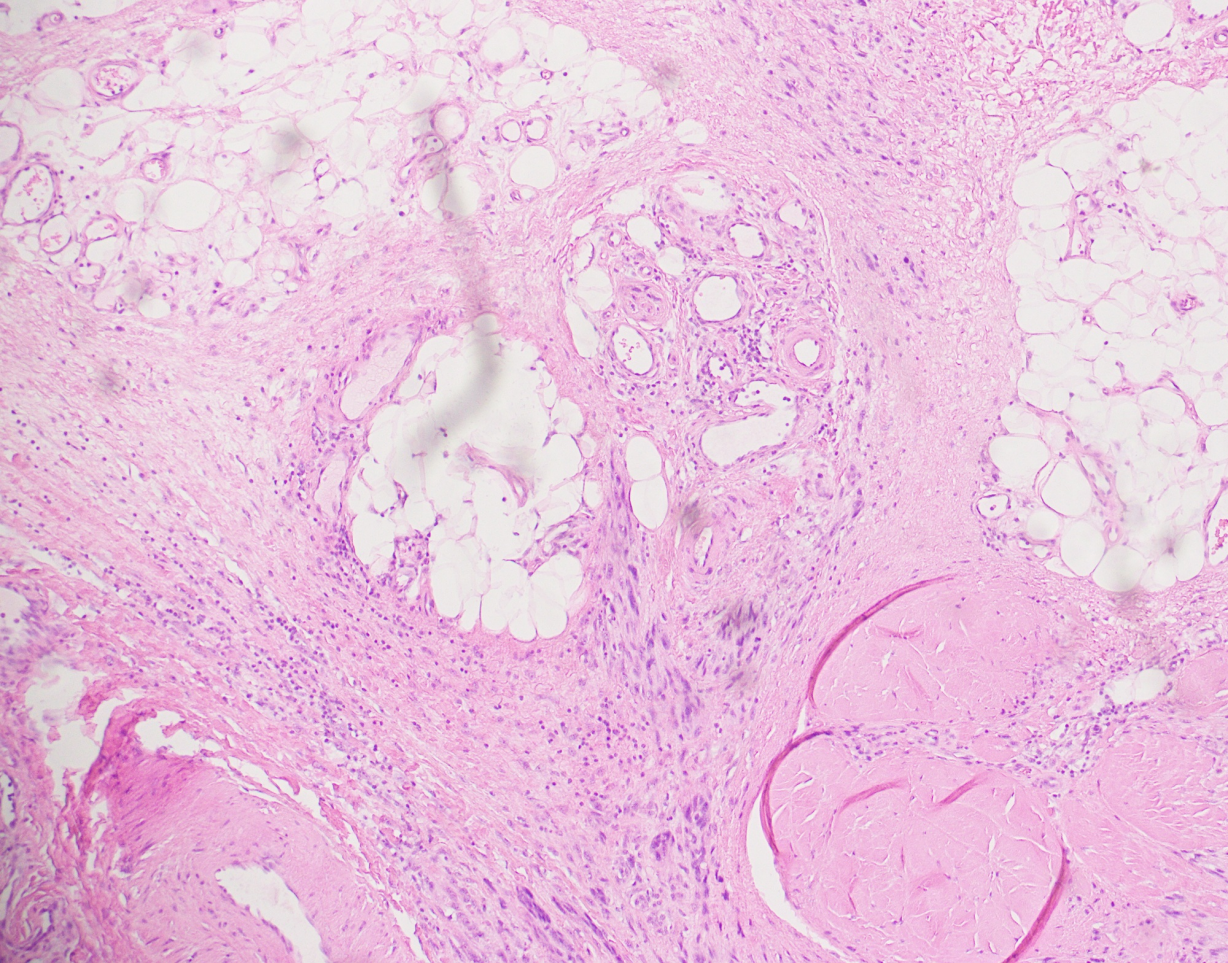
Tumor extending to Clark level V (involving the subcutaneous fat).

**Figure 3:**
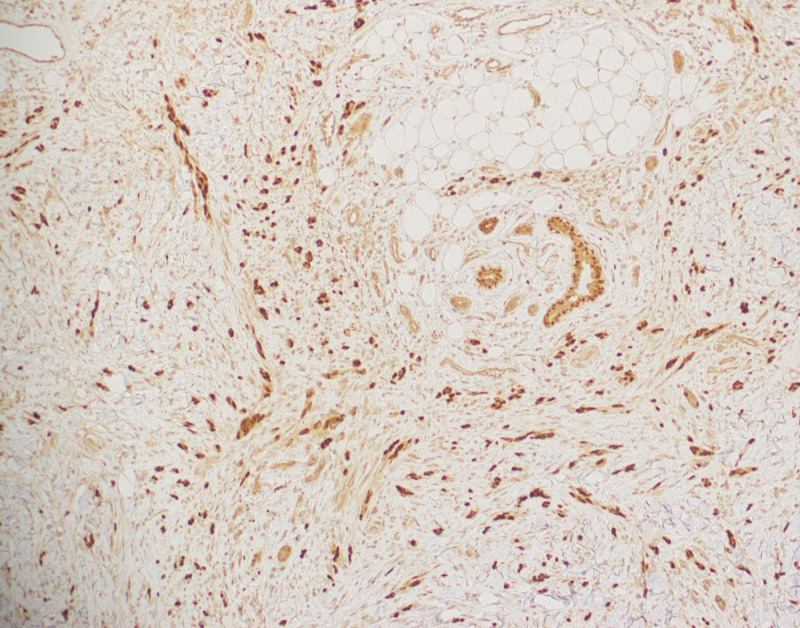
Nuclear staining with SOX10 immunohistochemistry highlights tumor cells extending into subcutaneous fat.

Regarding Clark level of invasion, 20 cases had invaded to level V at time of diagnosis, seven cases were class IV, two were class III, and one was class I. All of the level V cases exhibited pT4 level of invasion, while five level IV cases exhibited pT4, and two exhibited pT3 level of invasion. The two level III cases exhibited pT3 level of invasion, and the one level I case was pT1. Among level V cases, six exhibited pN0, five were pN1, four were pN2, and five were pN3. Four of the level IV cases were pN0, two were pN1, and one was pN2. The two level III cases were pN0, and one level I case was pN1. The two-year survival was 80% (16 patients) in level V cases, and 100% in levels IV and III. The one patient with level I disease died within two years. Ten, four, and one of levels V, IV, and III cases, respectively, developed distant metastasis. The mean survival duration for level V was 45 months, and 48 months for level IV. There was no statistically significant difference in the Clark levels in terms of two-year survival, development of metastasis, lymph node, and primary tumor staging according to chi-squared test (Figures 2 and 3).

No case exhibited tumor regression, and only one case exhibited a brisk lymphocytic response.

## Discussion

Among malignancies, melanoma currently ranks 19th overall and second among cutaneous malignancies [1]. The worldwide incidence of melanoma has increased over the past seven decades [2]. Studies in Pakistan have shown it to be the third-most common cutaneous malignancy after basal cell and squamous cell carcinoma [7,8]. Although third in incidence, it is the leading cause of mortality and morbidity among skin cancers [9,10]. Volumes of published studies have evaluated various prognostic parameters affecting outcomes in patients with cutaneous malignant melanoma; however, a review of the literature did not find any study involving the population of our country. 

A study by Ali et al. reported that the median age of patients with malignant melanoma is 57 years, which is younger than the median age at diagnosis of other solid malignancies [1]. Studies have shown that the most common sites of involvement of malignant melanoma are sun-exposed areas of skin [11,12]. In our study, sun-exposed areas were also commonly involved; however, an unusual point finding was the high incidence of melanoma in the anal regions (33%). This site has been found to be rarely involved in western published studies; however, our local epidemiological data also shows an increased incidence in the Pakistani population (14.2% compared to 0.2% in the west) [13,14]. Melanoma in the anal region has a comparatively dismal prognosis in terms of five-year overall survival as compared to melanomas of sun-exposed areas [15]. 

Breslow's thickness and Clark levels have remained a part of the pathology report of malignant melanoma for a number of years. The Breslow thickness contributes to staging of the primary tumor as per the TNM classification. A follow-up study of 3,323 patients of malignant melanoma was carried out at the John Wayne Cancer Institute in the United States by Morton et al. [16]. The study showed a fall in the five-year survival rate with increasing Breslow thickness (95% for <0.75 mm versus 46% for >4 mm) and Clark level (95% for level II versus 47% for level V). In a review of 371 patients of early-stage melanoma, Karakousis et al. observed a gradual decrease in five-year survival with increasing tumor thickness (a decrease of 3% with each millimeter up till 6 mm and a decrease of 8%/mm from 7 to 15 mm) [17]. Richetta et al. in a study of 21 patients conducted at the Sapienza University in Rome found increased Breslow thickness to be associated with increased risk of lymph node metastasis [18]. In comparison while other studies showed large numbers of early-stage melanomas, 80% of our cases (n = 25) were found to have advanced stage disease. 

Presence of nontraumatic ulceration is another important prognostic factor. It is included in the TNM staging of malignant melanomas. Egger et al. in a study of 736 patients found presence of ulceration and Breslow thickness > 2.3 mm to be independent risk factors for worse overall survival and disease free survival [19]. In our study, 60% of cases had ulceration, the presence of which affects primary tumor staging. We found that the presence of ulceration was associated with increased tumor thickness (60% of pT4 cases had ulceration); however, we did not find any statistically significant effect of ulceration on survival duration and metastasis. 

Our study compared with western data shows that most patients have locally advanced disease at time of diagnosis. This leads to increased morbidity and mortality. Most of the patients do not have adequate access to healthcare, which is enjoyed by the people of the developed countries. Majority of the population are residents of rural areas where the health facilities are in a miserable state. Besides this, there is lack of awareness regarding the disease leading to ignoring the early-stage lesions. Another factor contributing to the dismal prognosis is the increased incidence of anorectal melanomas that are known to have worse outcome as compared to melanomas of the sun-exposed sites. To gain further insight into the behavior of malignant melanoma in our population, it is important to have broader studies with early-stage melanomas and their follow-up. Gene mutation analysis may also provide help in understanding this aggressive malignancy and also identifying therapeutic targets. 

## Conclusions

Melanoma is an aggressive malignancy and often results in high morbidity and mortality. In our population, the disease is usually well-advanced at the time of diagnosis, leading to reduced survival. As such, broader studies with early-stage melanomas are needed to compare various prognostic factors and their effect on survival.
